# A comparison of the quality of image acquisition between the incident dark field and sidestream dark field video-microscopes

**DOI:** 10.1186/s12880-015-0078-8

**Published:** 2016-01-21

**Authors:** Edward Gilbert-Kawai, Jonny Coppel, Vassiliki Bountziouka, Can Ince, Daniel Martin, for the Caudwell Xtreme Everest and Xtreme Everest 2 Research Groups

**Affiliations:** University College London Centre for Altitude Space and Extreme Environment Medicine, UCLH NIHR Biomedical Research Centre, Institute of Sport and Exercise Health, 170 Tottenham Court Road, London, W1T 7HA UK; Statistical Support Service, Population, Policy and Practice Programme, Institute of Child Health, University College London, London, England; Department of Intensive Care, Erasmus MC University Hospital Rotterdam, 3000 Rotterdam, The Netherlands; Division of Neonatology, Erasmus MC-Sophia Children’s Hospital, Wytemaweg 80, P.O. Box 2060, 3000 CB Rotterdam, Netherlands

**Keywords:** Microcirculation, Microscopy, Validation, Capillary

## Abstract

**Background:**

The ‘Cytocam’ is a third generation video-microscope, which enables real time visualisation of the *in vivo* microcirculation. Based upon the principle of incident dark field (IDF) illumination, this hand held computer-controlled device was designed to address the technical limitations of its predecessors, orthogonal polarization spectroscopy and sidestream dark field (SDF) imaging. In this manuscript, we aimed to compare the quality of sublingual microcirculatory image acquisition between the IDF and SDF devices.

**Methods:**

Using the microcirculatory image quality scoring (MIQS) system, (six categories scored as either 0 = optimal, 1 = acceptable, or 10 = unacceptable), two independent raters compared 30 films acquired using the Cytocam IDF video-microscope, to an equal number obtained with an SDF device. Blinded to the origin of the films, the raters were therefore able to score between 0 and 60 for each film analysed. The scores’ distributions between the two techniques were compared.

**Results:**

The median MIQS (95 % CI) given to the SDF camera was 7 (1.5–12), as compared to 1 (0.5–1.0) for the IDF device (*p* < 0.0001). Of the six categories assessed by the MIQS, nearly one fifth of the SDF videos were scored as unacceptable for pressure (20 %), content (20 %), and stability (17 %), with focus scoring deficiently 13 % of the time. High agreement between the two raters scoring values was evident, with an intra-class correlation coefficient (ICC) of 0.96 (95 % CI: 0.94, 0.98).

**Conclusions:**

These results demonstrate that the quality of sublingual microcirculatory image acquisition is superior in the Cytocam IDF video-microscope, as compared to the SDF video-microscope.

**Electronic supplementary material:**

The online version of this article (doi:10.1186/s12880-015-0078-8) contains supplementary material, which is available to authorized users.

## Background

Incident dark field (IDF) imaging is an important technique that allows real time visualisation of the microcirculation [1]. Based upon the illumination of microvessels covered by a thin epithelial layer, it may be thought of as the successor to both orthogonal polarization spectroscopy (OPS) [2], and more recently, sidestream dark field (SDF) imaging [3]. Introduced in 2012, this third generation hand-held camera known as the Cytocam IDF video-microscope (Braedius Medical, Huizen, The Netherlands), was developed in an attempt to overcome many of the previous generations devices technical limitations [1]. These included; i) the limitations imposed by analogue video cameras, ii) the inability to achieve automatic microcirculation analysis, iii) pressure-induced microcirculatory alterations (predominantly caused by the heavy weight of the devices (SDF camera weight 320 g), iv) the requirement for hand operated focussing, and v) poor quality of image acquisition [4].

The Cytocam is a lightweight (120 g), fully digitalised pen-like device (length 220 mm, diameter 23 mm) that applies the principle of incident dark field microscopy introduced by Sherman and Cook in 1971 [5]. Blood vessels <100 μm in diameter, and <1000 μm below the surface of an organ or mucosal surface, are visualised in a two-dimensional plane through the process of epi-illumination [5]. Highly illuminating light emitting diodes (LEDs) enable suitable tissue penetration, and to avoid motion induced blurring secondary to fast moving erythrocytes [6], a very short LED pulse time of two milliseconds is utilised. Image delineation is optimised using a 3.5 megapixel high-resolution sensor, an optical magnification factor of four times, and an optical resolution of more than 300 lines/mm - an improvement of 50 % over SDF devices. This is further enhanced with an effective field of view (FOV) almost three times as large as earlier devices (1.55 × 1.16 mm, FOV area = 1.79 mm^2^), which may be magnified by a factor of 211 times on the display monitor [1]. Improved focussing is achieved through an integrated distance measurement system, which through the means of a manually adjusting the piezo linear motor via the computer interface, can alter the sensor position in steps of two microns. This novel quantitative focusing mechanism results in an accurate and repeatable focus distance, without having to repeatedly adjust the focus depth for every subsequent measurement. Finally, the IDF video-microscope has the capabilities for direct microcirculation analysis where the images are recorded digitally and analysed automatically. Specialised software automatically detects and quantitatively assesses the vessels’ diameters, and the flow velocity of erythrocytes within visualised vessels. Previously analysis of SDF videos required their conversion from analogue to digital images, with subsequent off-line analysis using specialised image processing software [7].

Although the IDF device should have significant superiority, in terms of image quality, over previous technologies this requires confirmation. We therefore set out to directly compare IDF and SDF images in a formalised manner.

## Methods

Thirty films of human sublingual microcirculation obtained using an SDF video-microscope (MicroVision Medical, Amsterdam, Netherlands), were compared to thirty comparable films obtained using the Cytocam IDF video-microscope. The films were picked at random from a database of over 800 SDF and IDF films, all of which were obtained from healthy adult volunteers who had given informed consent. Ethical approval for the study had been obtained from University College London Research and Ethics Committee. Two raters (EGK, JC), blinded to the device on which the video was generated, independently graded the films using the Microcirculation image quality score (MISQ) system [8]. In 2007 a consensus statement that outlined five key principles for optimal image acquisition [9]. These were:

1. Five separate image sites per organ
2. Avoidance of pressure artefacts
3. Elimination of secretions
4. Adequate focus and contrast adjustment
5. High quality recording

In 2013, a more formal approach to grading the quality of image acquisition prior to analysis was described, thereby giving a semi-objective measure of its suitability to be entered for computer analysis and quantification [8]. Six key characteristics of image capture were identified and encompassed within the ‘Microcirculation Image Quality Score’ (MIQS) (Table 1).

**Table 1:** The Microcirculation image quality score

Category	Brief description	Optimal (0)	Acceptable (1)	Unacceptable (10)
Illumination	Brightness and contrast of video	Even illumination across the entire field of view. Contrast sufficient to see small vessels against a background of tissue.	The video borders on being too dark or bright to distinguish vessels from tissue but the vessels are still identifiable.	The video is oversaturated/too bright or too dark to make out analysable features. Insufficient contrast to resolve flow rate.
Duration	Number of frames in the video clip and how it represents the actual pathology	Analysable video segment is ≥5 s long (>150 frames)	Analysable video segment is 3–5 s (between 90 and 150 frames)	Analysable video segment <3 s (90 frames)
Focus	Image sharpness in region of interest	Good focus for all vessels (small and large) in the entire field of view. Plasma gaps and red blood cells are visible.	<1/2 field of view is out of focus or edges of the vessels are slightly out of focus.	Video is completely out of focus such that no small vessel can be seen.
Content	Determination of the types of vessels and/or presence of occluding artefacts in the image.	Video is free of occlusions. Good distribution of large and small vessels. Less than 30 % of the vessels are looped upon themselves	Video may have a few artefacts. Acceptable distribution of large and small vessels. About 30–50 % of the vessels are looped.	Most of the field of view has occluding artefacts such as saliva or bubbles. More that 50 % vessels are looped upon themselves.
Stability	Frame motion that can be adequately stabilised without motion blur	Movement is within ¼ of the field of view. No motion blur.	Movement is within ½ field of view. No motion blur.	Movement is greater than ½ of the field of view and/or motion blur in frame
Pressure	Iatrogenic mechanical pressure causing misrepresentation of flow	Flow is constant throughout the entire movie. No obvious signs of artificially sluggish or stopped flow. Good flow in the largest vessels.	Signs of pressure (localised sluggish flow in a specific large vessel), but flow appears to be unimpeded based on good flow in most large vessels.	Obvious pressure artefacts associated with probe movement, and/or flow that starts and stops, reversal of flow. Poor or changing flow in larger venules.

Adapted from: ‘Quality Scoring Metrics: The microcirculation image quality score: development and preliminary evaluation of a proposed approach to grading quality of image acquisition for bedside videomicroscopy’ [8]

Each of the six categories is graded as 0 (optimal), 1 (acceptable) or 10 (unacceptable). If the total of the six categories is >10, then the video is unsuitable for Table 1 analysis and discarded. This somewhat peculiar scoring system is used, for if any one category is designated as unacceptable, it enforces that the video is not used [8].

The agreement between the two raters was assessed using the intra-class correlation coefficient and Bland and Altman limits of agreement. Over the range of scores given, Spearman’s correlation coefficient was used to assess the degree of over- or under- estimation of the score by either rater. The Mann–Whitney *U*-test was used to compare the score’s distribution between the two techniques. The two-tailed significance level was set at 0.05, and R (version 3.1.0) was used for the analyses.

## Results

**Fig. 1:**
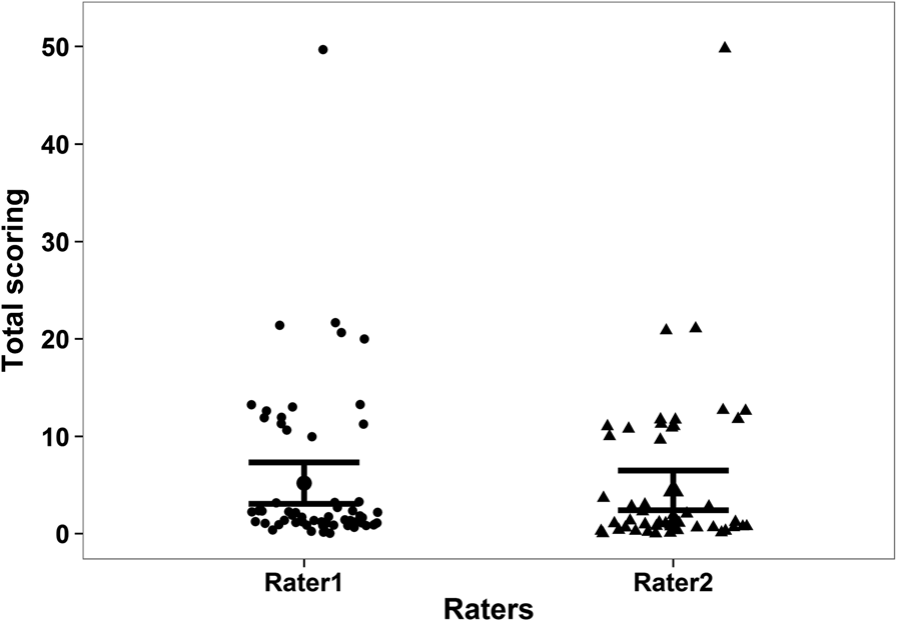
Distribution plot of total score values given by both raters with mean (95 % CI)

All 60 videos were analysed by both raters and no problems were encountered. The distribution of the individual total scores by rater is shown in Fig. 1.

**Fig. 2:**
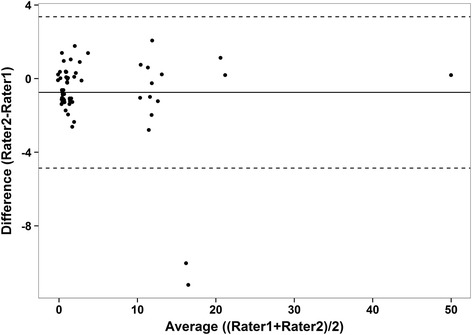
Bland Altman plot for the agreement between two raters

**Fig. 3:**
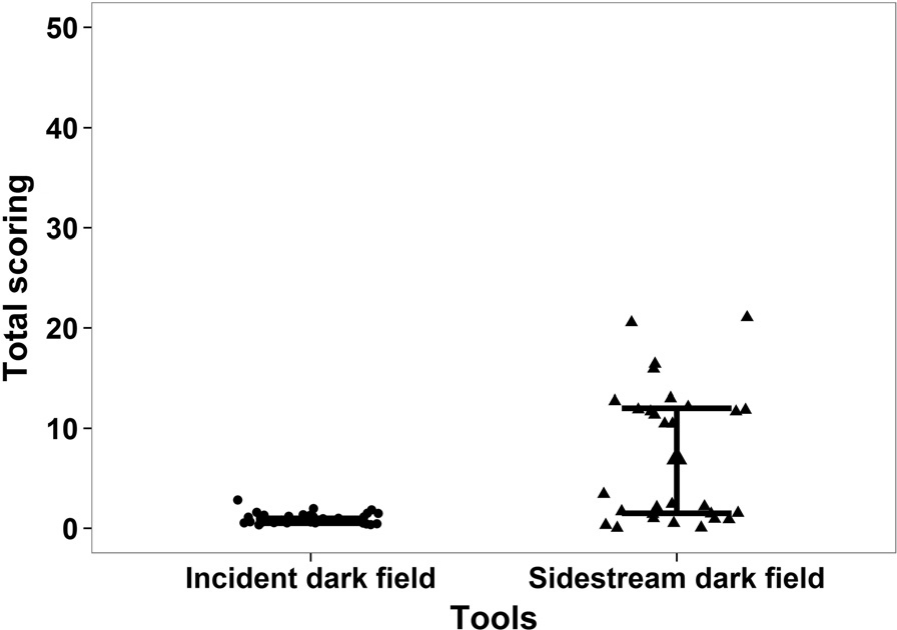
Distribution of scores according to the technique used to acquire the image with median (95 % CI)

Very good agreement between raters’ total scores are evident with an intra-class correlation coefficient of 0.96 (95 % CI: 0.94, 0.98). In addition, good agreement is evident in Fig. 2 (mean difference (rater2 – rater1): −0.75), and whilst some individual variation may exist as indicated by the slightly wide limits of agreement ( −4.86; 3.36), no over- or under- estimation trend by either rater was demonstrated (rho = −0.165, *p* = 0.21). For each device, the breakdown percentages of films scored as optimal, acceptable or unacceptable is presented in Table 2. When comparing the tools, the median score (95 % CI) given to the SDF video-microscope was 7 (1.5; 12), as opposed to 1 (0.5; 1.0) for the IDF video-microscope (*p* < 0.0001). The distribution of these values may be seen in Fig. 3. Examples of images taken with the Incident Dark Field imaging camera, and Sidestream Dark Field imaging camera can be seen in Additional files 1 and 2.

**Table 2:** Percentage (%) of IDF and SDF films scored as optimal, acceptable, or unacceptable

Category	Optimal (Score = 0)		Acceptable (Score = 1)		Unacceptable (Score = 10)	
	IDF	SDF	IDF	SDF	IDF	SDF
Stability	68	50	32	33	0	17
Pressure	73	67	27	13	0	20
Illumination	92	67	8	30	0	3
Duration^a^	100	100	0	0	0	0
Focus	98	43	15	43	0	13
Content	88	65	12	15	0	20

^a^Images were all cut to 150 frames in length prior to analysis, hence both IDF and SDF demonstrate optimal scores for this category

## Discussion

These results demonstrate for the first time, that the Cytocam IDF video-microscope is superior to the SDF video-microscope in terms of the quality of sublingual microcirculatory image acquisition.

High agreement between the two raters scoring values was demonstrated, and whilst it is evident from the Bland Altman plot that some individual variation existed between raters, neither individual demonstrated a trend in over- or under-estimating the score as the total values increased. Using the total score value to determine if an image was deemed suitable for analysis, (i.e. if given a total score ≥10 renders the video as unacceptable), there was 100 % exact agreement (95 % CI: 94 %; 100 %) between the two raters.

As to whether the IDF video-microscope was superior to the SDF video-microscope in terms of providing acceptable images for analysis, the median score of 7 given to the SDF images, as opposed to 1 for the IDF videos, indicates that the SDF camera is more prone to produce unacceptable results. In this instance, 100 % of the images obtained using the IDF video-microscope were judged to be acceptable for data analysis, as opposed to only 50 % of these data collected using the SDF device. Table 2 demonstrates how the individual components of the MIQS system were scored for both cameras. From this we are able to see which categories SDF scored particularly poorly for as compared to IDF. The IDF video-microscope did not receive any scores of 10 from either rater, however nearly a fifth of the SDF videos were scored as unacceptable for stability (17 %), pressure (20 %), and content (20 %), with focus scoring deficiently 13 % of the time. This indicates superior IDF image acquisition for multiple categories, as opposed to in only one area of data capture.

Although 60 films chosen at random from a large database of images were analysed (30 for each device), a weakness in this manuscript was that no power calculation was performed prior to commencing. This said, the strong statistical significance supports the belief that it was adequately powered. Additionally, as the MIQS still relies on observer input to grade images, it is thus subjective in its film assessment. Nevertheless, it is the most formal approach to image grading we have to date, and the high ICC supports its use.

## Conclusion

In conclusion, these data demonstrate that the IDF video-microscope provides improved image acquisition of human sublingual microcirculation when compared to the SDF video-microscope. Superior in five out of the six categories comprising the MIQS, the use of IDF offers an advanced insight into the clinical evaluation of the microvasculature.

## Additional files

Additional file 1:
**Two examples of images obtained using the incident dark field video-microscope.** (PNG 1659 kb) Additional file 2:
**Two examples of images obtained using the sidestream dark field video-microscope.** (PNG 1188 kb)

## Abbreviations

FOV: Field of view
IDF: Incident dark field
LED: Light emitting diode
MIQS: Microcirculation image quality score
OPS: Orthogonal polarization spectroscopy
SDF: Sidestream dark field
