# Supplementary figures and images for: A comparison of the quality of image acquisition between the incident dark field and sidestream dark field video-microscopes
Source: BMC Med Imaging. 2016 Jan 21;16:10. doi: 10.1186/s12880-015-0078-8 (PMC4722634; doi:10.1186/s12880-015-0078-8)

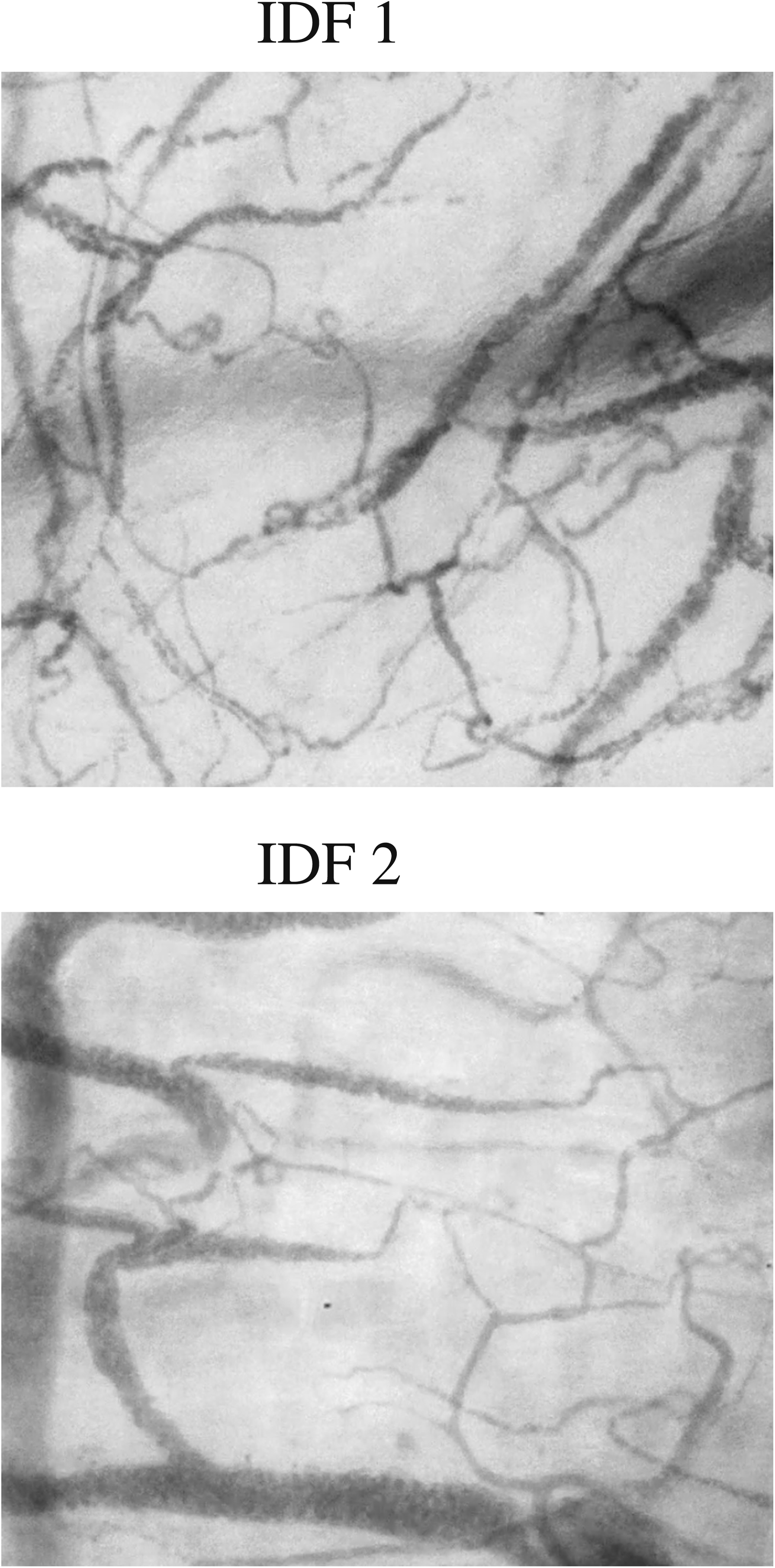

Supplement: Additional file 1: — Two examples of images obtained using the incident dark field video-microscope. (PNG 1659 kb) [file 12880_2015_78_MOESM1_ESM.png]

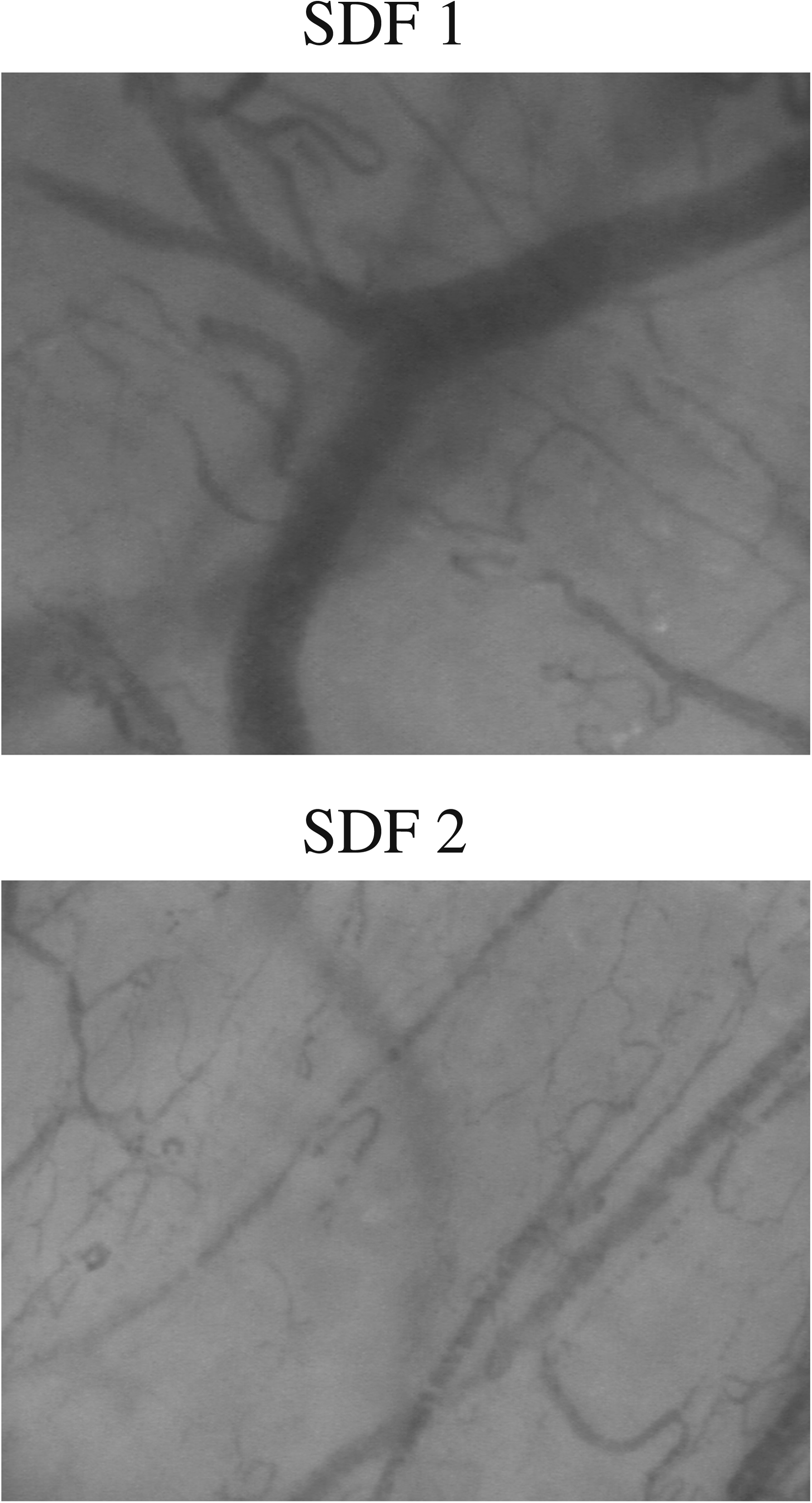

Supplement: Additional file 2: — Two examples of images obtained using the sidestream dark field video-microscope. (PNG 1188 kb) [file 12880_2015_78_MOESM2_ESM.png]
